# Primary bacterial ventriculitis in adults, an emergent diagnosis challenge: report of a meningoccal case and review of the literature

**DOI:** 10.1186/s12879-018-3119-4

**Published:** 2018-05-18

**Authors:** Anaïs Lesourd, Nicolas Magne, Anaïs Soares, Caroline Lemaitre, Muhamed-Kheir Taha, Isabelle Gueit, Michel Wolff, François Caron

**Affiliations:** 1 Infectious Disease, Rouen University Hospital, Rouen, France; 2 Radiology, Rouen University Hospital, Rouen, France; 3 Microbiology, Rouen University Hospital, Rouen, France; 4 Intensive Care, Rouen University Hospital, Rouen, France; 5 Institut Pasteur, National Reference Centre for Meningococci and Invasive Bacterial Infections Unit, Paris, France; 6 Infectious Disease, Bichat-Claude Bernard University Hospital, Paris, France; 7 CHU Charles Nicolle-Service de Maladies infectieuses et Tropicales, 1 rue de Germont, 76000 Rouen, France

**Keywords:** Meningococcal meningitis, Ventriculitis, Levofloxacin, Cerebral vasculitis, Diagnosis, Magnetic resonance imaging

## Abstract

**Background:**

Defined by an infection of the ventricular system of the brain, ventriculitis is usually known as a health-care associated infection. In contrast, primary pyogenic ventriculitis complicating community-acquired meningitis is uncommon, and mainly described in infants. Only seven cases that have occured in adults have been found in the international literature.

**Case presentation:**

We report here a new case due to *Neisseria meningitidis* occurring in an 85 year-old-man. The comparison with previous reports allows to drawn several conclusions: (i) cases occurred in relatively old adults (median age: 65 years); (ii) *Streptococcus pneumoniae*, *N. meningitiditis* an*d Staphylococcus aureus* are the leading responsible pathogens; (iii) atypical clinical presentation seems the rule in which meningism often lacks; (iv) in absence of clinical or biological specific parameters, modern brain imaging such as magnetic resonance imaging with gadolinium enhancement is of utmost importance for the diagnosis, leading to anticipate an increase of the diagnosis in the near future, thanks to easier access to such exploration; (v) death or serious sequelae commonly occurred; (vi) prolonged antibiotic courses (6 weeks to 3 months) have been used, without strong rational. In the given case, the patient presented with a lack of meningeal irritation signs. The diagnosis was made by MRI considering a lasting confused state. A four-week antibiotic regimen was successful, combining two weeks of intravenous cefotaxime followed by two weeks of oral levofloxacin much easier to administrate and allowing early rehabilitation.

**Conclusion:**

Primary bacterial ventriculitis is a real diagnosis challenge. Larger indications of MRI for bacterial meningitis, particularly in cases with an atypical presentation or poor evolution would certainly increase the number of diagnosis.

## Backgound

Ventriculitis most commonly occurs as a complication of external ventricular drains or in patients with ventricular shunts used to relieve increased intracranial pressure associated with hydrocephalus. Such infections are not uncommon (infection rate of ventricular-catheter raising up to 20% in some series) and are caused by microorganisms involved in foreign body infections such as staphylococci or antibiotic resistant Gram-negative bacilli [1–3]*.*

In contrast, only few cases of primary ventriculitis have been reported, most of them being group B streptococci neonatal infections [4–6]. In a 2017 review by Gronthoud et al. of primary ventriculitis, only six cases have been described in adults including only one due to *Neisseria meningitidis* [7]*.* While such infections are supposed to complicate meningitis, surprisingly no meningism was reported in five out of these six cases, despite meningism being an important clinical feature in the diagnosis of meningitis.

We herein report a new case of meningococcal ventriculitis occurring in an elderly and enabling to discuss the interest of fluoroquinolones in such setting.

## Case report

The patient was an 85 year-old-man, with a previous history of atrial fibrillation requiring a long-term curative anticoagulant therapy, renal lithiasis and benign prostatic hyperplasia. He was addressed to the emergency department for a fall in a context of fever at home. As he was afebrile at his arrival in hospital with no evident diagnosis, no antibiotic was introduced. Two days later the patient’s condition worsened with high fever (39.4 °C), confusion and altered mental status, without any neck stiffness nor other symptom of meningism. A computed tomography (CT) scan without contrast showed no abnormality. A lumbar puncture revealed a purulent cerebrospinal fluid (CSF) with 5220 white-cells per mm^3^ (82% of neutrophils) and rare cocci of undetermined Gram staining on direct microscopic examination. Intravenous antibiotic therapy with cefotaxime and oxacillin was immediately started in combination with adjunctive IV dexamethasone (10 mg q 6 h). Due to the installation of a comatose state (Glasgow coma score 9/15), the patient was admitted to the intensive care unit, intubated and mechanically ventilated.

After 24 h, the CSF’s culture yielded a group B *N. meningitidis* strain with decreased susceptibility to penicillin (MIC = 0.125 mg/L for penicillin G and = 0.250 mg/L for amino penicillin, MIC = 0.003 mg/L for cefotaxime, MIC = 0.002 mg/L for ciprofloxacin). Cefotaxime alone was continued at 200 mg/kg/day combined with dexamethasone during the first four days (both according to current national guidelines for bacterial meningitis in adults [8]).

**Fig. 1:**
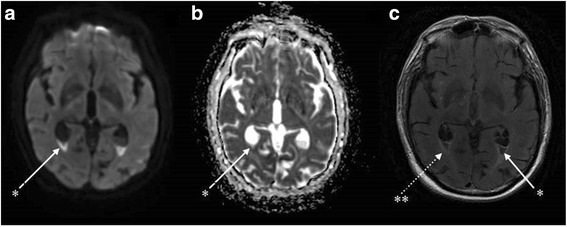
Brain magnetic resonance imaging (day 6) showing an aspect of ventriculitis in multiple axial sequences. *Irregular declivous intraventricular debris with restricted diffusion in the occipital horns with a moderate dilatation of the lateral ventricles seen in all sequences: diffusion (**a**), apparent diffusion coefficient (**b**) and T2 fluid attenuated inversion recovery (FLAIR) gadolinium (1c). **Periventricular hyperintensities and ependymal enhancement in the axial T2 FLAIR with gadolinium enhancement sequence (**c**)

**Fig. 2:**
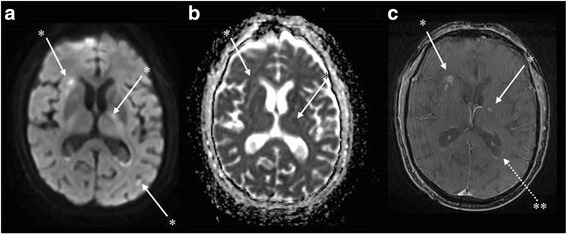
Brain magnetic resonance imaging (day 6) showing possible cerebral vasculitis in multiple axial sequences. *Punctiform hyperintensities in diffusion-weighted imaging (**a**) in several vascular territories with cortical and sub-cortical distribution. These lesions appear with a normal or decreased axial apparent diffusion coefficient value (**b**) and some are enhanced after gadolinium injection in T1 sequences suggesting a blood-brain barrier disruption (**c**) and consistent with semi-recent ischemic lesions. **Ependidymal enhancement in T1 with gadolinium enhancement sequences, sign of ventriculitis (**c**)

The patient’s neurological state progressively improved to regain a vigilant state and was extubated on day 5 of antibiotic therapy. He was discharged to the infectious diseases unit. Nonetheless, he remained confused and somnolent (Glasgow coma score 14/15). Magnetic resonance imaging (MRI) with T2 fluid-attenuated inversion recovery (FLAIR) sequences performed on day 6 revealed the presence of a declivous purulent material inside the lateral ventricles with a moderate dilatation of these structures, not requiring any drainage (fig. 1). This material showed restricted diffusion (decreased apparent diffusion coefficient [ADC] value) and no magnetic susceptibility artefacts on T2* sequences, ruling out the possibility of a blood sediment. It also revealed punctiform hyper intensities in T2 weighted sequences and b1000 diffusion-weighted imaging (DWI) in multiple vascular territories with cortical and deep white matter distribution. These lesions were characterised by a normal or decreased ADC value and some of them were enhanced after gadolinium injection (fig. 2). All of these findings were consistent with semi-recent ischemic strokes.

**Fig. 3:**
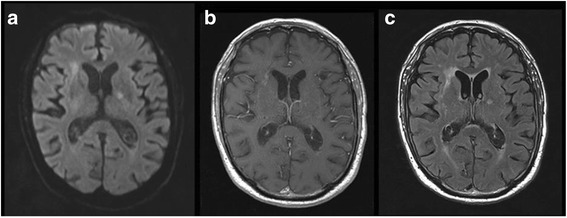
Brain magnetic resonance imaging at four weeks of treatment in axial multiple sequences. Decrease of the level of debris layering the occipital horns of the lateral ventricles in all sequences: axial diffusion b1000 (**a**), axial T1 gadolinium (**b**) and axial T2 fluid attenuated inversion recovery (FLAIR) gadolinium sequences (**c**). Clear decrease of the periventricular hyperintensities and ependymal enhancement in T1 with gadolinium (**b**) and T2 FLAIR gadolinium (**c**) sequences. Decrease of b1000 DWI hyperintensities. No apparition of new ischemic lesions

Because of this unfavourable outcome, the patient received a prolonged duration of antibiotic therapy, namely cefotaxime for 2 weeks switched to oral levofloxacin (500 mg q 24 h) for another 2-week period. After four weeks of antibiotic treatment, the MRI showed a decrease of the purulent debris inside the ventricles with a clear attenuation of the signal (fig. 3). At that time the patient had regained a normal vigilant status. He only suffered from a cerebellar syndrome with a gait disturbance attributed to ischemic cerebellar lesions. After three months of follow-up, the patient’s condition had improved, allowing him to walk and to return home, even if the help of a wheelchair was sometimes required.

## Discussion and conclusions

Before the antibiotic era, pathological studies of patients who died from bacterial meningitis have shown that the ventricular fluid usually turned cloudy by the end of the first week of the infection [9]. Nowadays, the incidence of this complication is considered as very low. However, among the six primary ventriculitis cases recently reported by Gronthoud et al.*,* four have been diagnosed during the last ten years [7]. Easier access to modern brain imaging such as MRI has certainly played a role and will increase the number of diagnosis in the near future. MRI including gadolinium-enhanced sequences is the best imaging, particularly in T2 FLAIR sequences which reveal periventricular hyperintensity, an ependymal enhancement and irregular intraventricular debris layering in the occipital horns [10, 11]. The presence of ventricular debris is reported in 16 out of 17 cases (94%) of healthcare-associated ventriculitis described by Fukui et al. [11]. According to these authors, an irregular intraventricular debris is quite specific for pus and helps differentiating from a straight level of acute clotted blood [11]. MRI is preferred to a CT-scan, which lacks sensitivity, can mislead the clinician to a diagnosis of intracerebral bleeding or can miss the diagnosis [7]. Thus, MRI should be considered in those patients who fail to improve despite appropriate antibiotic therapy.

Interestingly, adding the current case to the seven found in the literature (the 6 from Gronthoud et al. and one case reported in Japanese language [4]), the median age was of 65 years (rank of 39–85), our patient being the oldest.

Neck stiffness is present in 74% of patients with bacterial meningitis [12]. However, five out of the six patients reviewed by Gronthoud et al. as well as our patient did not have this sign. While it is well established that some meningococcal meningitis might have an atypical presentation, especially those caused by serogroup W [13], this seems quite the rule in primary pyogenic ventriculitis.

**Fig. 4:**
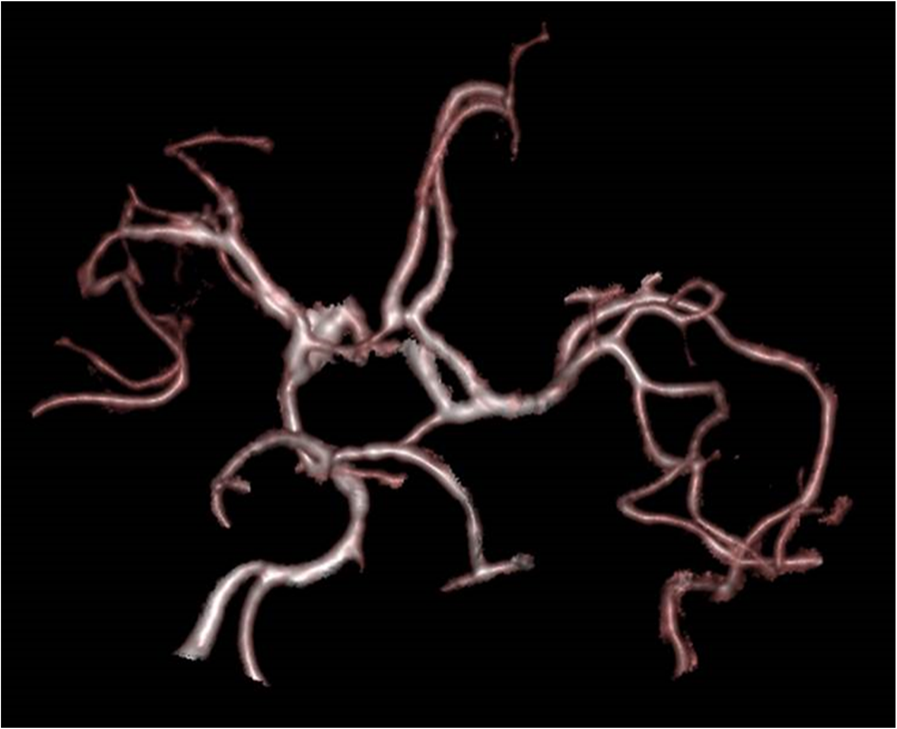
Magnetic resonance imaging vascular volume rendering reconstruction obtained from the 3D-T1 gadolinium sequences. Absence of proximal vascular stenosis nor thrombosis

Our patient had multiple ischemic brain lesions located in various vascular territories, which may correspond to cerebral vasculitis. Routine MRI protocol procedures initially performed did not include angiographic sequences. Retrospectively, volume rendering (VR) 3D-vascular reconstructions were performed with the T1 gadolinium sequences revealing no sign of proximal vascular stenosis nor thrombosis (fig. 4). Considering the patient was under curative anticoagulant therapy (for his atrial fibrillation), embolic strokes were here ruled out and infectious vasculitis was considered as highly probable. Such complication is relatively common during the course of pneumococcal meningitis (incidence of 9.8% in a retrospective multicentric study of 162 patients [14]). In contrast, to our best knowledge, no case of meningococcal cerebral vasculitis in adults has been published so far, while one case has been recently reported in a child [15].

While there are precise guidelines for the management of ventricular-catheter related infections [16], we found no recommandations nor expert advice for the management of the treatment of primary bacterial ventriculitis concerning neither the optimal regimen nor its duration. Among the previous cases, precise data on antibiotic treatment are given for only 4 of them: one of the two meningococcal cases was treated for 6 weeks (ceftriaxone 2 g q 12 h) [7] and the other for 3 months (ceftriaxone for 17 days followed by 12 weeks of oral moxifloxacin), both of them recovering without any sequelae [4]; a patient with *Streptococcus intermedius* ventriculitis received a 6-week-combination of cefotaxime and metronidazole [17] and a methicillin-resistant *S. aureus* case received an 8-week course of vancomycin (5 days) switched to oral linezolid (49 days) [18].

A 6 to 12-week duration of treatment is similar to what is commonly recommended for brain abscesses [19] despite the absence of evidence to consider this length essential in ventriculitis (for which the bacterial density as well as the antibiotic diffusion are potentially less problematic). However, given the severity of the disease, a long duration of antibiotic therapy could be considered providing the tolerance is acceptable.

We chose an initial regimen of third generation cephalosporin secondarily switched to a fluoroquinolone. Even if the patient did not experience any adverse event with cefotaxime, the goal was to shorten the IV treatment duration. Indeed, this oral switch allowed the patient to regain a faster functional autonomy once released from a parenteral perfusion. Levofloxacin, like other fluoroquinolones, achieves good concentrations into the CSF. It was here preferred to moxifloxacin, ensuring a better tolerance particularly in terms of epilepsy and cardiac toxicity [20, 21].

In the absence of cerebral suppurative lesions, the treatment was stopped after a total of 4 weeks. This relatively short treatment duration was validated by the absence of relapse.

Finally, our patient received adjunctive dexamethasone according to current guidelines, i.e., as soon as possible when the lumbar puncture reveals a purulent CSF [22]. Such a treatment has been proven to be beneficial in preventing hearing loss and neurological sequelae in adult purulent bacterial meningitis, particularly those due to *Streptococcus pneumoniae* [22, 23]. In the current case, dexamethasone however did not prevent the evolution to a ventriculitis probably due to a late diagnosis and treatment initiation in this elderly patient with an initial atypical presentation.

In conclusion, larger indications of MRI for bacterial meningitis, particularly in cases with an atypical presentation or poor evolution, would certainly increase the number of diagnosis of primitive pyogenic ventriculitis as well as of cerebral vasculitis in the near future. For susceptible strains, fluoroquinolones regimens are an attractive antibiotic class allowing an oral easily-to-tolerate treatment, even if strong evidence for long treatment is lacking.

## Abbreviations

ADC: Apparent diffusion coefficient
CSF: Cerebrospinal fluid
CT scan: Computed tomography scan
DWI: Diffusion-weighted imaging
FLAIR: Fluid-attenuated inversion recovery
MIC: Minimal inhibitory concentration
MRI: Magnetic resonance imaging
*N. meningitidis*: *Neisseria meningitidis*
